# Research and Optimization of Surface Roughness in Milling of SLM Semi-Finished Parts Manufactured by Using the Different Laser Scanning Speed

**DOI:** 10.3390/ma13010009

**Published:** 2019-12-18

**Authors:** Andrzej Matras

**Affiliations:** Production Engineering Institute, Mechanical Faculty, Cracow University of Technology, 31-155 Kraków, Poland; amatras@mech.pk.edu.pl; Tel.: +48-12-374-3250

**Keywords:** face milling, surface roughness, SLM, AlSi10Mg

## Abstract

The paper studies the potential to improve the surface roughness in parts manufactured in the Selective Laser Melting (SLM) process by using additional milling. The studied process was machining of samples made of the AlSi10Mg alloy powder. The simultaneous impacts of the laser scanning speed of the SLM process and the machining parameters of the milling process (such as the feed rate and milling width) on the surface roughness were analyzed. A mathematical model was created as a basis for optimizing the parameters of the studied processes and for selecting the sets of optimum solutions. As a result of the research, surface with low roughness (Ra = 0.14 μm, Rz = 1.1 μm) was obtained after the face milling. The performed milling allowed to reduce more than 20-fold the roughness of the SLM sample surfaces. The feed rate and the cutting width increase resulted in the surface roughness deterioration. Some milled surfaces were damaged by the chip adjoining to the rake face of the cutting tool back tooth.

## 1. Introduction

The Additive Manufacturing (AM) technology, commonly known as 3D printing, allows to layer-by-layer parts manufacture. One of the AM variants is Selective Laser Melting (SLM) which can be an alternative for casting of metals. It involves using a laser beam for selective melting of metal powders of which a part is made. This technology gives new possibilities to manufacture machine parts, but is not flawless. The diagnosed flaws include porosity, incomplete powder melting, insufficient dimensional, and shape accuracy and high surface roughness [1,2,3,4,5].

The process parameters have a principal impact on the quality and properties of parts manufactured using the SLM method. The basic parameter that characterizes the SLM process is the energy density of laser beam. The laser beam energy density increases with the laser power increase, decrease of the scanning speed, decrease of the powder layer thickness, or decrease of the hatching space [6,7]. As the laser beam energy density increases, the density of parts made in the SLM process initially grows and then is reduced [7,8]. The increase of the laser beam energy density causes the temperature increase which results in the low viscosity and a large amount of liquid metal [1,9]. The temperature increase prolongs the residence time of the molten pool as a result of which the metal flows easily and fills the pores [10]. When the laser energy input is too low, large-sized irregular pores form due to the small amount of liquid and the high viscosity as well as the short lifetime of the molten pool. Excessively high laser beam energy density causes vaporization of the material, creating spherical pores [11]. The surface roughness of the SLM-made parts, in correlation to the porosity, initially decreases and then grows as the laser beam energy density increases [9,12]. 

Many authors deal with the experimental research in order to optimize the SLM process parameters and the melting of AlSi10Mg powder. The research aims at obtaining the parts of high density, low porosity, low surface roughness, and good mechanical properties. Wei et al. [13] analyzed the impact of scanning speed and laser power. While applied high scanning speeds, it is observed an increased number of pores, microstructure defects and unmolten powders. An analogous scanning speed impact was also observed elsewhere [9,12]. Maamoun et al. [14] obtained a greater density of the parts by combining higher scanning speed, larger laser power and lesser hatching space, or by combining lower scanning speed and moderate laser power. Analogously as Gao et al. [15], they observed that the microhardness of the samples measured along the x-z plane increases as the laser beam energy density decreases. The authors concluded that the optimum laser beam energy density range for melting the AlSi10Mg powder is 50–60 J/mm^3^ as it minimizes the occurrence of pores in the structure. Trevisan et al. obtained the analogous analysis results [16].

The surface roughness of SLM-manufactured components is insufficient because of the requirements for contemporary machine parts. For this reason, a two-stage approach is sometimes used that involves a finish machining of SLM-manufactured components [17,18,19]. There is, however, a small number of publications in the literature on the machining of SLM-manufactured semi-finished parts. By means of turning, Kaynak and Kitay [19] reduced the surface roughness of an SLM-manufactured semi-finished part made of the 316L stainless steel. Struzikiewicz et al. [17] during the turning of an SLM-manufactured semi-finished part also made of the 316L stainless steel came to the conclusion that it is the feed rate that has the largest impact on the surface roughness. Analogous analysis results were obtained by [18] after the milling of an SLM-manufactured semi-finished part made of CM-Ni-Cu powder and by [20] after the turning of an SLM-manufactured semi-finished part made of the Inconel 718 alloy powder. The authors did not analyze, however, the impact of the SLM process parameters.

Milton et al. [21] studied the orientation, determined by the system of coordinates of the SLM machine in which a SLM semi-finished part was manufactured using the Ti6Al4V alloy powders. The authors studied the surface roughness obtained at stage two, after the milling. The least roughness was obtained after machining the x-z plane, the largest on the x-y plane. They did not analyze, however, the impact of the SLM process parameters and the milling on surface roughness. 

There have been also the studies comparing the machinability of materials manufactured traditionally and in the SLM process. Struzikiewicz et al. [22] analyzed the turning of the AlSi10Mg alloy and noticed many burrs and breaches on the machined surfaces of SLM-manufactured semi-finished parts. During the machining with various cutting parameters, decrease of surface roughness values were obtained on the traditionally manufactured semi-finished part. Dumas et al., on the other hand [23], milled and polished SLM semi-finished parts made of the Ti6Al4V alloy. By using the subtractive material removal process, the authors reduced the surface roughness, but did not notice significant differences between the SLM and traditionally manufactured semi-finished parts. The authors did not analyze the impact of the SLM process parameters either.

Additive manufacturing has many advantages which can be used for manufacturing of semi-finished parts. By using additive techniques, it is possible to obtain parts with complex geometries. This can be used to produce geometrically complex semi-finished parts, what requires only applying subtractive finishing machining without roughing. It is also possible to cut only some surfaces that are required to have enough surface roughness that cannot be obtained by using additive techniques. The semi-finished parts with a complex shape are not necessarily purposed to be mass-produced, as in case e.g., forging. The literature review indicates a lack of publications which analyze simultaneously the impact of the SLM process parameters and the subsequent machining on the obtained surface roughness. It is obvious that a simultaneous optimization at the material production and processing stages can provide benefits in the form of better optimization of results. Therefore, the paper includes a simultaneous analysis and optimization of the selected parameters of the SLM and milling process. The used optimization criterion is Ra and Rz surface roughness parameters. 

Section 1 includes an analysis of the current knowledge. Section 2 gives the characteristics of the AlSi10Mg alloy and describes the research methodology. Section 3 analyses the experimental results, creates the mathematical model and selects the sets of optimum solutions. Section 4 includes the summary and conclusions.

## 2. Materials and Methods 

The research includes the milling of semi-finished parts manufactured using the SLM technology. The SLM process was performed on a TruPrint 1000 machine (Trumpf, Ditzingen, Germany) with a 200 W fiber laser. The SLM parts were made of the powders of the AlSi10Mg alloy which is widely used in the automotive and aerospace industries. The powder grain sizes are given in Table 1 and the chemical composition of powder in Table 2.

The SLM process parameters were chosen based on the literature review. The laser scanning speed *v* was variable in the 600–1400 mm/s range which allowed obtaining the laser beam energy density *E_d_* of 31–73 J/mm^3^. The remaining, constant SLM process parameters are presented in Table 3.

**Figure 1:**
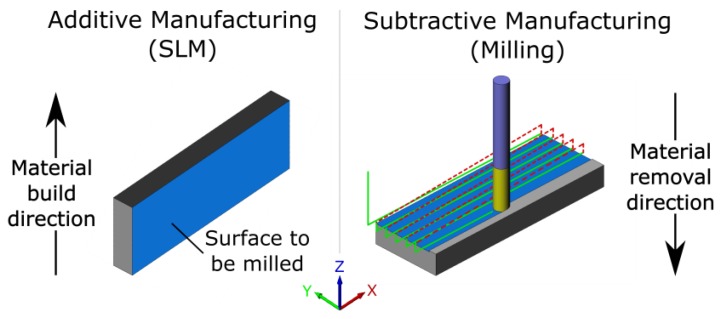
Schematic diagram of the Selective Laser Melting (SLM) and milling processes.

The SLM process was used to manufacture semi-finished parts of the following dimensions: 45 mm in axis x, 5 mm in axis y, and 15 mm in axis z. Based on the analysis results of authors [21], the SLM semi-finished parts were subjected to milling on the x-z plane (Figure 1).

The machining was performed on a CNC MiniMill2 machine tool (Haas, Oxnard, CA, USA) using a double-edged end mill cutter with 3 mm diameter and catalogue number 0068030A (Datron, Mühltal, Germany). The cutting tools with small diameter are more universal. These cutting tools give the possibility to perform pocket bottom with walls characterized small inner radius. The tool is characterized in Table 4. The machining zone was lubricated with an oil mist. 

The machining parameters were chosen based on the recommendations of the cutting tool manufacturer. The feed rate *f* was variable in the 835–2045 mm/min range, and the machining width *a_e_*, varied from 0.829 to 1.67 mm. The machining depth was constant and equal to *a_p_* = 0.1 mm and the machining speed was *v_c_* = 848 m/min. A pneumatic spindle type 602CAT40 (Air Turbine Technology) with rotating speed of *n* = 90,000 rpm was used in the milling process. Up milling was analyzed.

The roughness was measured on a Talysurf Intra 50 profilometer (Taylor Hobson, Leicester, UK). To perform the surface roughness measurements, a measuring tip with a rounding radius of 2 μm was used. The measurements were performed in the transverse direction to the machining marks (parallel to the cutting tool feed). The Ra and Rz parameters were determined in accordance with ISO 4287. After surface roughness measurements, non-periodic primary profiles were obtained. The *λ_c_* filter value was selected based on the values recommended for non-periodic profiles. Based on the obtained ranges of the surface roughness parameters values, the cut-off values *λ_c_* = 0.8 mm and *λ_s_* = 2.5 μm were selected. The measurements were repeated five-fold. The microscopic observations of the surface were performed by using the VHX-600 digital microscope (Keyence, Osaka, Japan) at 500× magnification.

The studies were conducted based on a central-compositional design of experiment. The variables were analyzed on five levels. The measurement analysis results were examined on the Statistica software (13.1, StatSoft, Tulsa, OK, USA) for the *α* = 0.05 significance level.

## 3. Results and Discussion

### 3.1. Impact of the Laser Scanning Speed and Machining Parameters on the Surface Roughness

The surfaces of manufactured semi-finished parts were subjected to roughness measurements. The best surface roughness (Ra = 3.55 ± 0.32 μm, Rz = 18.3 ± 2.3 μm) was obtained for a semi-finished part made with the lowest laser scanning speed *v* = 600 mm/s which corresponds to the laser beam energy density of *E_d_* = 73 J/mm^3^. The surface roughness parameter increases with increasing the laser scanning speed. For highest laser scanning speed *v* = 1400 mm/s (*E_d_* = 31 J/mm^3^) the values of roughness parameters were Ra = 6.91 ± 0.65 μm and Rz = 38.3 ± 3.9 μm, respectively. The higher standard deviations of the surface roughness parameters are observed for the higher laser scanning speeds, which indicates the lower homogeneity of the surfaces.

**Figure 2:**
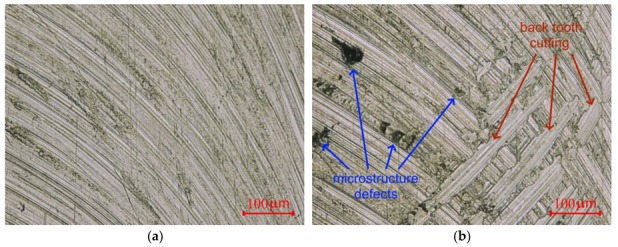
Photographs of the milled surfaces with applied the laser scanning speed and feed rate, respectively: *v* = 600 mm/s, *f* = 835 mm/min (**a**) and *v* = 1400 mm/s, *f* = 2045 mm/min (**b**); the constant cutting width *a_e_* = 1.25 mm.

After milling of the SLM semi-finished parts, microscopic observations of the obtained surfaces were performed. Microscopic photographs of the milled surfaces of SLM semi-finished parts produced by using the various values of laser scanning speed *v* and, the next, milled with using the various values of feed rate *f* were presented in Figure 2a,b.

The photographs present surface fragments made during the last pass of the cutting tool. Therefore, only the traces made during the last tool pass are visible, not erased by the traces from successive passes, which occurs during the face milling.

On same samples, defects are observed in the internal microstructure of the SLM semi-finished parts. The amount of defects increases with increasing the laser scanning speed. Analogous results were also observed in the previous papers [9,12,13,24].

The surface damages due to the cutting tool back tooth were observed on the surfaces. The probable mechanism of this phenomenon takes place in result adjoining chip to the rake face of cutting tool after the machining cycle is completed. Then, the chip moves under the cutting tooth and damages the surface. Such phenomena are observed also during the machining of other Al alloys. The analysis of the samples indicates that lower values of feed rate and lower values of laser scanning speed minimize the surface damage resulting from the cutting tool back tooth. The differences between the surfaces made using various milling widths are insignificant. 

Based on the measurements of surface roughness parameters, the ANOVA was performed and the regression equations were determined. The ANOVA was performed in order to determine the significance of the impact of the analyzed process parameters on the surface roughness. The results of the ANOVA analysis for Ra and Rz roughness parameters are given in Table 5 and Table 6, respectively. The process parameters which affect the surface roughness at the applied significance level of *α* = 0.05 are marked in bold typeface.

The regression Equations (1) and (2) can be expressed as follows:

Ra = (97.435 × 10^−6^)*v* + (24.434 × 10^−5^)*f* − (53.592 × 10^−9^)*f*^2^ + (14.598 × 10^−3^)*a_e_* − 77.844 × 10^−3^ (1)

Rz = (43.281 × 10^−5^)*v* + (49.716 × 10^−5^)*f* + (19.939 × 10^−2^)*a_e_* + 20.324 × 10^−2^ (2)

The obtained regression equations indicate a good fit between the calculated and the measured values. The R^2^ is 0.87 for parameter Ra and 0.78 for parameter Rz, respectively.

**Figure 3:**
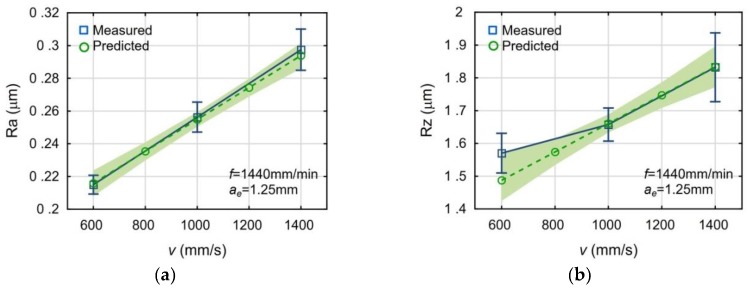
Impact of the laser scanning sped *v* on the surface roughness parameters: Ra (**a**) and Rz (**b**).

**Figure 4:**
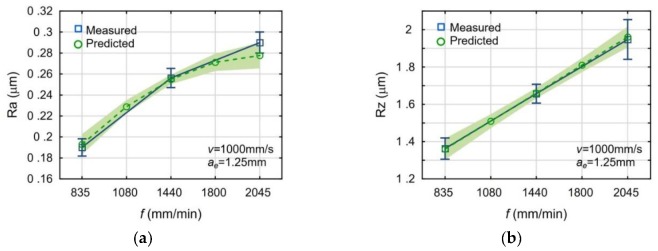
Impact of the feed rate *f* on the surface roughness parameters: Ra (**a**) and Rz (**b**).

**Figure 5:**
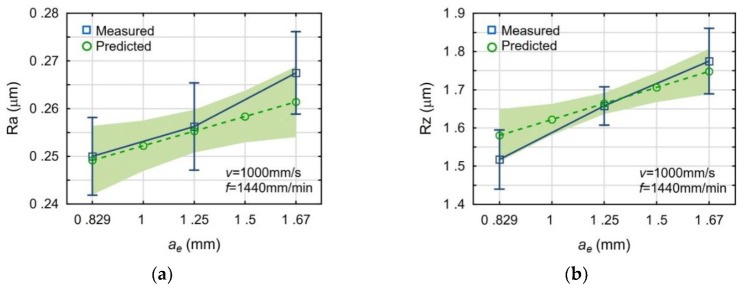
Impact of the cutting width *a_e_* on the surface roughness parameters: Ra (**a**) and Rz (**b**).

Figure 3, Figure 4 and Figure 5 present the impact of the analyzed process parameters on the Ra and Rz surface roughness parameters. The mean values and standard deviations of the measured surface roughness parameters were marked with blue color. The predicted values and the prediction ranges were marked with green color. All mean measured values are in the ranges of prediction. The maximum deviation, between the mean values of measured and calculated, is below 6%.

The increase of the laser scanning speed and the feed rate deteriorates the surface roughness of the milled surfaces. The increased milling width also deteriorates the surface roughness, but the trend is less significant in this case. The observed influences of the machining parameters are typical for the face milling.

### 3.2. Application of the Response Surface Methodology for the Optimization of the Analysed Process Parameters

**Figure 6:**
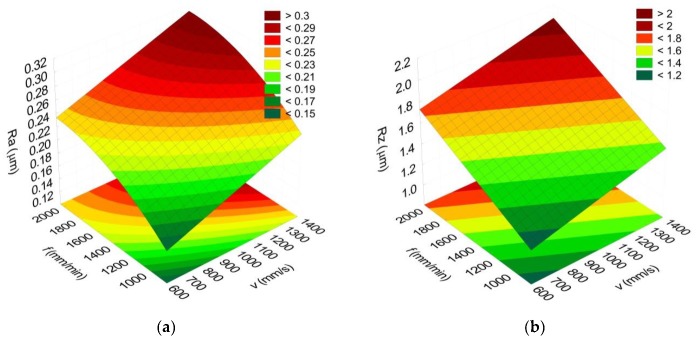
Impact of the laser scanning sped *v* and the feed rate *f* on the surface roughness parameters: Ra (**a**) and Rz (**b**); *a_e_* = 1.25 mm.

The Response Surface Methodology (RSM) was used to optimize the parameters of the studied processes. The RSM is widely used in the optimization of technological processes. The mathematical model was created for significant factors determined based on the ANOVA. Figure 6 presents the impact of the laser scanning speed *v* and the feed rate *f* on the Ra and Rz parameters for the milling width of *a_e_* = 1.25 mm.

Based on the mathematical model (Equations (1) and (2)), the least surface roughness was calculated for the lowest process values of *v* = 600 mm/s, *f* = 835 mm/min, and *a_e_* = 0.829 mm. The calculated values of surface roughness parameters in this case are Ra = 0.142 ± 0.013 μm and Rz = 1.043 ± 0.094 μm. Surface roughness measurements were made in order to verify the model, and the measured mean values of the surface roughness parameters values were Ra = 0.144 ± 0.008 μm and Rz = 1.104 ± 0.089 μm.

**Figure 7:**
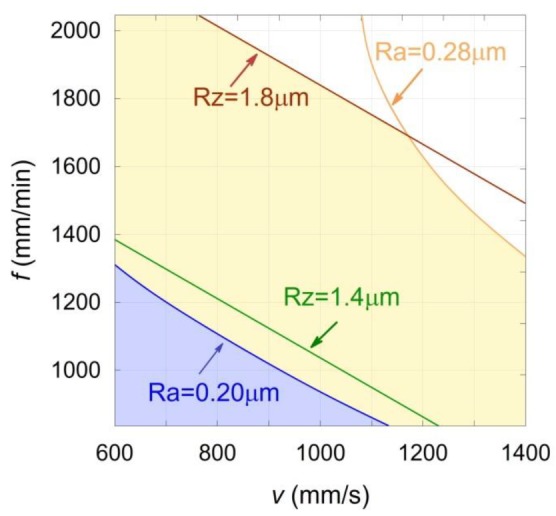
Diagram for the selecting optimum laser scanning speed *v* and the feed rate *f* at a constant cutting width *a_e_* = 1.25 mm designed for specified case I and II surface roughness parameters.

A surface of the lowest possible roughness is not always desirable. The specification of machine parts includes the limit values of surface roughness. The Ra and Rz values were specified for further optimization of the surface roughness parameters, as follows: for case I Ra ≤ 0.2 μm and Rz ≤ 1.4 μm, and for case II Ra ≤ 0.28 μm and Rz ≤ 1.8 μm. As the analysis covers the parameters of two different processes, the optimum solutions can be obtained in many ways. The diagram for the selecting optimum laser scanning speed and the feed rate at a constant cutting width *a_e_* = 1.25 mm are presented in Figure 7. The ranges of variable values of the analyzed process parameters were marked with blue area for case I and orange area for case II.

Based on Figure 7, it is possible to choose one optimum solution, but the features of the manufactured part must be known. The choice of the optimum solution depends, inter alia, on the volume of the material which is added while the additive manufacturing and removed while the subtractive manufacturing. Without any additional assumptions, at this stage it is not possible to choose one optimum solution. Thus, only the relationship between the optimum laser scanning speed and the feed rate was chosen.

## 4. Conclusions

The mathematical model created with the use of RSM allows a prediction of the surface roughness parameters based on the values of the studied SLM and milling processes. As a result of using the model, the process parameters were chosen which enabled to obtain a surface with the roughness of Ra = 0.14 μm and Rz = 1.1 μm, respectively. The sets of optimum process parameters were also chosen, as a result of which it is possible to obtain the surface roughness of Ra ≤ 0.2 μm and Rz ≤ 1.4 μm or Ra ≤ 0.28 μm and Rz ≤ 1.8 μm, respectively (Figure 7). It addition, it was found that:

1. The milling allows the surface roughness of the SLM-manufactured semi-finish parts to be reduced more than 20-fold.
2. The impact of the laser scanning speed on the surface roughness of the SLM-manufactured semi-finish parts was observed. The increase of the laser scanning speed in the analyzed range causes a deterioration of the surface roughness of SLM-manufactured semi-finished parts.
3. Defects in the internal microstructure of the SLM semi-finished parts manufactured by using high laser scanning speed were observed.
4. The impact of the laser scanning speed used in the SLM process on the obtained by milling surface was observed. The milled SLM semi-finish parts made at higher laser scanning speeds have higher surface roughness. It was also observed that the chip adjoining to the rake face of back cutting tooth causes the milled surface damages. This phenomenon intensifies as the laser scanning speed used in the SLM process increases.
5. The impact of the studied milling parameters on the milled surface roughness was observed. The surface roughness increases as the feed rate grows. The increase of cutting width also increases the surface roughness, but to a lesser degree than the feed rate increases.

## Figures and Tables

**Table 1:** Particle size distribution.

D10	D50	D90
21.4 µm	33.7 µm	54.0 µm

**Table 2:** Chemical composition of AlSi10Mg.

Element	Fe	Si	Mg	Mn	Zn	Cu	Other	Al
**Composition, (wt.%)**	0.14	10.4	0.33	<0.01	<0.01	0.03	<0.04	Balance

**Table 3:** The SLM process parameters.

Parameter	Value
Laser power (W)	175
Hatching space (μm)	200
Powder layer thickness (μm)	20
Scan Strategy	Chessboard
Shielding gas	Ar
Diameter of laser beam (μm)	30
Laser wavelength (nm)	1.070
Chamber temperature (°C)	26.8 (27% RF)
Oxygen level (%)	0.11

**Table 4:** The geometry of the milling tool.

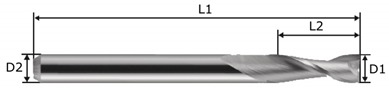				
D1	D2	L1	L2	Helix Angle
3 mm	3 mm	40 mm	10 mm	30°

**Table 5:** ANOVA for surface roughness parameter Ra.

Source	Adj SS	DF	Adj MS	F-Value	*P*-Value
***v***	**0.025450**	**1**	**0.025450**	**144.7667**	**0.000000**
*v* ^2^	0.000180	1	0.000180	1.0251	0.315513
***f***	**0.038103**	**1**	**0.038103**	**216.7457**	**0.000000**
***f*^2^**	**0.002637**	**1**	**0.002637**	**14.9982**	**0.000276**
***a_e_***	**0.000779**	**1**	**0.000779**	**4.4314**	**0.039625**
*a_e_* ^2^	0.000238	1	0.000238	1.3515	0.249785
*v·f*	0.000132	1	0.000132	0.7519	0.389443
*v· a_e_*	0.000004	1	0.000004	0.0217	0.883412
*f· a_e_*	0.000135	1	0.000135	0.7687	0.384241
Residual Error	0.010196	58	0.000176		
Total	0.077778	67			

**Table 6:** ANOVA for surface roughness parameter Rz.

Source	Adj SS	DF	Adj MS	F-Value	*P*-Value
***v***	**0.493185**	**1**	**0.493185**	**37.6595**	**0.000000**
*v* ^2^	0.000093	1	0.000093	0.0071	0.933264
***f***	**1.924128**	**1**	**1.924128**	**146.9261**	**0.000000**
*f* ^2^	0.020342	1	0.020342	1.5533	0.217653
***a_e_***	**0.145135**	**1**	**0.145135**	**11.0825**	**0.001519**
*a_e_* ^2^	0.030916	1	0.030916	2.3607	0.129860
*v·f*	0.011284	1	0.011284	0.8617	0.357123
*v·a_e_*	0.020938	1	0.020938	1.5988	0.211132
*f·a_e_*	0.004389	1	0.004389	0.3352	0.564870
Residual Error	0.759561	58	0.013096		
Total	3.462712	67			
